# Reply to ‘Comment on Age of enlightenment: long-term effects of outdoor aesthetic lights on bats in churches' by T. Onkelinx

**DOI:** 10.1098/rsos.171630

**Published:** 2017-12-20

**Authors:** Jens Rydell, Johan Eklöf, Sonia Sánchez-Navarro

**Affiliations:** 1 Biology Department, Lund University, 223 62 Lund, Sweden; 2 Graptolit Ord och Natur, Krokdalsvägen 88, 517 34 Bollebygd, Sweden; 3 Estación Biologica de Doñana CSIC, C/Americo Vespucio 26, 41092 Sevilla, Spain

We are happy to learn that the basic statistic that we employed in our study [1] was correct and provided essentially the same result as the modern and much more sophisticated methods presented by Thierry Onkelinx in his comment [2]. We highly appreciate his effort and trust that his statistics were carefully done and provided clear and robust results, thereby certainly increasing the power of our findings.

We would like to point out, however, that we deliberately made our paper as easy to read as possible and we avoided complex calculations on purpose. By using simple statistics that anybody may understand and perhaps even repeat, we believe that the accessibility of our study to other than professional biologists and statisticians was increased. The decision to publish in an open access journal aimed at the same goal.

We believe that simplicity may sometimes have a value on its own in, e.g. applied studies, as long as the scientific quality is not compromised. In our case, the avoidance of complex statistics most likely makes the study more frequently read, and, in particular, more often consulted by those that we hope will take action in the conservation issue addressed [1]. In our view, there is no reason to make things more complicated than necessary, and this may be the case even if the intended audience consists of the scientific community alone.

## Data accessibility

This article has no additional data.
